# MicroRNAs in Obesity and Related Metabolic Disorders

**DOI:** 10.3390/cells8080859

**Published:** 2019-08-09

**Authors:** Jean-François Landrier, Adel Derghal, Lourdes Mounien

**Affiliations:** Aix Marseille Univ, INSERM, INRA, C2VN, 13005 Marseille, France

**Keywords:** micro-RNA, obesity, diabetes, adipose tissue, pancreas, inflammation, leptin

## Abstract

Metabolic disorders are characterized by the inability to properly use and/or store energy. The burdens of metabolic disease, such as obesity or diabetes, are believed to arise through a complex interplay between genetics and epigenetics predisposition, environment and nutrition. Therefore, understanding the molecular mechanisms for the onset of metabolic disease will provide new insights for prevention and treatment. There is growing concern about the dysregulation of micro-RNAs (miRNAs) in metabolic diseases. MiRNAs are short non-coding RNA molecules that post-transcriptionally repress the expression of genes by binding to untranslated regions and coding sequences of the target mRNAs. This review aims to provide recent data about the potential involvement of miRNAs in metabolic diseases, particularly obesity and type 2 diabetes.

## 1. Introduction

Gene expression can be controlled at a transcriptional level by the activity of DNA-binding transcription factors or controlled post-transcriptionally by changes in RNA stability or localization, protein translation, or biological half-life. The microRNAs (miRNAs) play predominantly inhibitory regulatory roles by binding to cis-elements in the 3′ untranslated region (3′UTR) of message-encoding RNAs. The miRNAs are small noncoding RNA molecules of 21 to 25 nucleotides that regulate gene expression [1]. They were first discovered in *Caenorhabditis elegans* in 1993 and, later on, in vertebrates and plants [2,3]. Today thousands of miRNAs have been identified, showing them to be one of the most abundant classes of gene-regulatory molecules in multicellular organisms. These noncoding RNAs behave as specific gene silencers by base pairing to 3′UTR of target messenger mRNAs, but have also been proved to bind anywhere along the length of the mRNA transcript to exert their effects. miRNAs exert their actions by inhibiting translation and by affecting mRNA stability and degradation [1,4]. Most mammalian miRNAs are transcribed by RNA polymerase II as long precursor molecules, including stem-loop structures. This primary transcript is cleaved by the complex containing the RNase III-type enzyme Drosha and the DGCR8/Pasha protein yielding a ≈ 70 nucleotide hairpin-structured precursor (pre-miRNA) [5]. The pre-miRNA is transported into the cytoplasm and cleaved by another RNase III enzyme called Dicer to generate a ≈ 22 nucleotide mature miRNA (functional) [6]. Upon separation of the 2 strands, the guide strand binds to an Argonaute (Ago) protein and is integrated into the RNA-induced silencing complex (RISC) allowing the identification of complementary sites within the 3′UTRs of target mRNAs [6]. Based on computational algorithms, around 60% of human transcripts contain potential miRNA-binding sites within their 3′UTRs [7]. It is also useful to note that miRNAs can also bind to the 5′ untranslated (5′UTR) and coding sequences of the mRNA [8,9,10]. A single miRNA potentially has the ability to bind to more than 100 target mRNAs, and multiple miRNAs can cooperate to finely tune the expression of the same transcript [11,12,13]. MiRNAs play key roles in numerous physiological processes, including cell proliferation, apoptosis, neurodevelopment, and tissue differentiation but also in pathological processes [14,15].

Interestingly, several miRNAs have recently been found to regulate adipose tissue biology (development and metabolism), insulin secretion and action, and therefore their imbalance may play a role in the development of obesity and related metabolic complications [16,17,18]. For instance, miR-14, miR-278 and let-7 are involved in the metabolism of lipid and glucose [19,20]. Although the miRNAs are involved in several diseases, for the purpose of this review, we have focused our attention on the functions of miRNAs in two aspects of the metabolic diseases, that is, obesity and type 2 diabetes (T2D). The PubMed and Web of Science databases were used to search for relevant published literature in the field of miRNAs and metabolic disorders. The few core keywords used were: micro-RNA, diabetes, obesity, inflammation, pancreas, adipose tissue, insulin, leptin, adipogenesis, metabolic syndrome.

## 2. MiRNAs and Metabolic Diseases

### 2.1. MiRNAs, Obesity and its Metabolic Complications

Obesity and overweight are two very common diseases in our modern societies. According to the World Health Organization (fact sheet n°311), worldwide obesity rates, measured by the body mass index (BMI), have nearly doubled since 1980. In 2008, 35% of adults were overweight and 11% were obese. More than 40 million children under the age of five were overweight in 2011. While this phenomenon is well known in United States, with an adult obesity rate of 35%, overweight and obesity are expanding massively in other industrialized and developing countries [21]. The spread of obesity is also associated with a real public health problem because of its costs and health effects. Indeed, excess body weight increases the likelihood of various metabolic diseases such as heart disease, T2D, dyslipidaemia, osteoarthritis, and certain types of cancer [22].

Obesity and overweight usually result from an imbalance between energy intake and energy output. They are caused by the interaction of multiple factors, such as caloric and food intake, physical inactivity, genetic predisposition and individual metabolism. Essential treatment of obesity consists of low-calorie low-fat diets, increased physical activity, and diverse strategies contributing to the modification of lifestyle. However, weight losses achieved with lifestyle intervention are modest and limited by high rates of recidivism and compensatory slowing metabolism. In this context, it is critical to understand the contribution of the genetic and epigenetic traits in the onset of obesity. In accordance with this fact, different studies have suggested that multiple loci on the human genome are associated with obesity and metabolic syndrome [23,24,25]. Interestingly, omics approaches implied a correlation between the expression of several miRNAs in different tissues (e.g., adipose tissue, liver and pancreas) and obesity or metabolic diseases [24,26,27]. In the study by Kunej et al., among 1736 loci associated with obesity, 221 correspond to micro-RNAs [24]. In addition, different studies reported that the expression of miRNAs directly correlated with diet and lifestyle [28,29]. Although the list of miRNAs associated with a diet is important, several studies suggest that the miR-17/20/93 family, miR-21/590-5p family, miR-200b/c family, miR-221/222 family, let-7/miR-98 family and miR-203 are the most dysregulated in this context [28].

The control of energy homeostasis is finely tuned by endocrine and neural mechanisms that cooperate to maintain the balance between caloric intake and energy expenditure [30,31]. In this respect, the central nervous system (CNS) continuously monitors modifications in metabolic parameters (i.e., blood glucose or free fatty acids levels) or hormones (insulin, leptin and ghrelin) and elicits adaptive responses like food intake regulation or autonomic nervous system modulation [30,31]. Particularly, leptin is an adipose-derived hormone that is crucial to maintaining both normal body weight and insulin sensitivity by acting in the different nuclei of the hypothalamus as arcuate or ventromedial nuclei [32,33,34]. In addition, obesity is associated with inflammation in adipose tissue. The production of inflammatory cytokines can interfere with insulin signaling and can then contribute to T2D, as well as many other obesity-related diseases [35,36]. In this context, the endocrine function of the adipose tissue is crucial to maintain a normal weight and the regulation of energy homeostasis. Subsequently, it is not unexpected that miRNAs may be a new layer of regulation of the different functions of the adipose tissue in obesity [37].

Many miRNAs are differentially regulated in white adipose tissue of obese subjects compared to non-obese human Individuals [38,39,40,41]. Adipose tissue from the visceral area is more important for the metabolic aspects than the subcutaneous tissue [42]. In this context, profiling analysis showed that numerous miRNAs are expressed differently in subcutaneous and visceral white adipose tissue [43]. In humans, several studies have illustrated a correlation between the expression of miRNAs in adipose tissue and different metabolic parameters (BMI, adipogenesis, glycemia, leptinemia) [38,44]. In accordance with this observation, Heneghan et al. discovered that the expression of miR-17-5p and miR-132 differed significantly between obese and non-obese omental fat [45]. Interestingly, the expression of these two miRNAs in omental fat and blood from obese patients correlated significantly with glycosylated hemoglobin, leptin, body mass index and fasting blood glucose [45]. An increase in the expression of miR-21 was found in the white adipose tissue (WAT) of obese humans compared to lean controls, and it was positively correlated with BMI [46].

Recently, the group of J.W. Helge measured the miRNA expression in subcutaneous adipose tissue from 19 individuals with severe obesity (10 women and 9 men) before and after a 15-week weight loss intervention [47]. This intervention led to up-regulation of miR-29a-3p and miR-29a-5p and down-regulation of miR-20b-5p [47]. It has been highlighted in a recent study that the expression levels of miR-221 are up-regulated in obese people and that this miRNA can modulate fat metabolism through leptin and tumor necrosis factor-α (TNF-α) [48]. Interestingly, additional groups also reported an increase in the expression of miR-221 in the adipose tissue and liver from leptin deficient ob/ob and diet-induced obese (DIO) mice [48,49]. Other studies suggested that the expression of a large number of miRNAs is modified in mouse models of obesity [50]. For instance, Chartoumpekis et al. demonstrated the up-regulation of miR-342-3p, miR-142-3p, miR-142-5p, miR-21, miR-146a, miR-146b, miR-379 and the down-regulation of miR-122, miR-133b, miR-1, miR-30a, miR-192 and miR-203 during the development of obesity in mice [51]. The different mouse models for obesity (DIO or ob/ob) can be used as relevant models to test the involvement of one miRNA in obesity. As mentioned above, miR-21 is up-regulated in obese humans [46]. In a novel approach, the group of S. Dimmeler showed that locked nucleic acid (LNA)-miR-21 treatment led to significant weight loss and reduced adipocyte size, as well as repression of targets such as TGFβ-receptor 2 (TGFBR2) and phosphatase and tensin homolog (PTEN) [52]. In the same vein, let-7 knockout mice did not develop insulin resistance despite diet-induced obesity [19]. Interestingly, several studies have shown that weight loss modulated the circulating levels of miRNAs [53,54]. For instance, Manning et al. found that several miRNAs showed plasma levels comparable to those in lean controls after acute weight loss in women who are obese [53].

**Figure 1:**
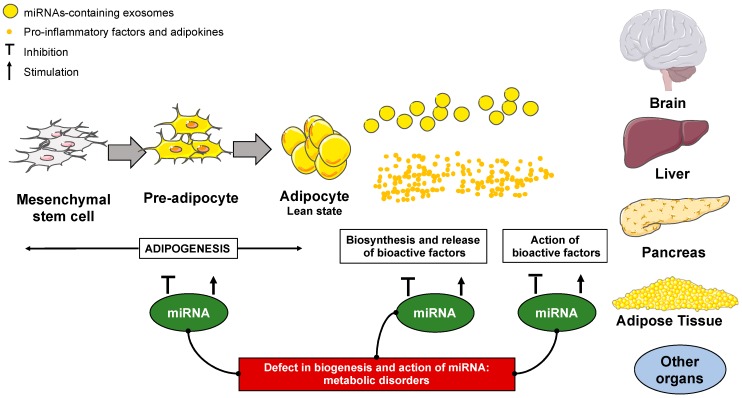
The functions of micro RNAs (miRNAs) in the adipose tissue development and functions. miRNAs regulate adipogenesis, the release and the action of the signals (adipokines, inflammatory factors and exosomes) derived from adipose tissue.

Endogenous miRNAs produced within adipocytes have been well-characterized. However, recently an increasing number of exogenous miRNAs have been proved to exist in different biological fluids, such as plasma, serum, urine and saliva [55,56]. Many of these fluid-based miRNAs are present in small extracellular vesicles, called exosomes, that are secreted by various cell types including adipose cells. Adipose tissue can release exosomal miRNAs that can act as signaling molecules [57] (Figure 1). In the context of obesity, DIO or ob/ob mice released more exosomes than control mice [58]. By this mechanism, adipose tissue can modulate the expression of genes located in other metabolic organs (Figure 1). For instance, the group of R. Kahn nicely demonstrated that exosomal miRNAs released from adipose tissue affected the hepatic expression of fibroblast growth factor 21 (FGF-21) involved in glucose homeostasis [59]. Altogether, these different studies suggest that miRNAs can be used as biomarkers in the context of metabolic diseases, but they could be also targeted for pharmaceutical approaches.

Collectively, these different facts highlighted the interest in the role of miRNAs in the field of obesity and related diseases. Particularly, the alteration in miRNA expression could induce changes in the pattern of genes controlling a range of biological processes including adipogenesis as well as inflammation of the adipose tissue, lipid metabolism and insulin resistance. In this part of the chapter, we will mainly evoke aspects linked to the functions of miRNAs in the biology of the adipose tissue and the pancreas that are interdependent for lipid and glucose metabolism.

### 2.2. The Role of MiRNAs in Adipogenesis

A better understanding of the regulation of adipogenesis is essential for the development of new therapies against the onset of obesity. In fact, mild obesity is paired with an increase of adipose cell size (hypertrophic obesity), while severe obesity or childhood obesity also involves an increase of the number of adipocyte (hyperplasia obesity). The adipogenesis appears as a complex process involving several hormones or transcription factors as peroxisome proliferator-activated receptor γ (PPARγ), CCAAT/enhancer-binding protein (C/EBP) family and Sterol regulatory element-binding protein 1 (SREBP1) [60]. Recently, numerous studies exhibit that miRNAs play an important role in adipocyte differentiation and contribute to the development of obesity (Figure 1). The effect of miRNAs on adipogenesis happens to be complex as there are multiple targets for miRNAs. Nevertheless, miRNAs regulate adipogenesis by modulating the expression of key factors of adipocyte differentiation [61,62].

The first evidence suggesting a role for miRNAs in adipogenesis was in *Drosophila* flies illustrating that miR-14 and miR-278 regulate lipid metabolism [63,64]. Among the several miRNAs, special attention has been given to miR-143. Using antisense oligonucleotides transfected into human preadipocytes, miR-143 was identified as a regulator of adipocyte differentiation [65]. In accordance with this study, Takanabe et al. highlighted that miR-143 levels in adipose tissue of obese mice closely correlated with expression levels of adipocyte differentiation markers such as PPARγ [66]. Another example is miR-130a that inhibits adipogenesis through the down-regulation of the expression of PPARγ [67]. In the context of adipogenesis, Wang et al. demonstrated that the miRNA cluster miR-17-92, including miR-17, miR-18a, miR-19a, miR-19b-1, miR-20a, miR-92a, is up-regulated during adipocyte clonal expansion and accelerated adipocyte differentiation by negatively regulating the key cell cycle regulator and tumor suppressor gene Rb2/p130 [68]. As mentioned above, various miRNAs can affect adipogenesis by targeting C/EBP. For instance, miR-375 promotes 3T3-L1 adipocyte cell line differentiation by up-regulating the expression of C/EBP and PPARγ2 [69]. In another set of experiments, a correlation between miR-519d and the protein levels of PPARα involved in fatty acid homeostasis has been demonstrated [39]. The miR-519d suppressed the translation of the PPARα protein, and increased lipid accumulation during preadipocyte differentiation [39].

### 2.3. The Adipokines and MiRNAs

Adipose tissue is an important endocrine organ known to secrete nearly 600 adipokines that could be used as biomarkers in the clinical field [70]. The most important adipokines include leptin, adiponectin, resistin, apelin [71]. These adipokines exert a large range of effects on diverse organs indicating that the impairment of adipose tissue function alters the normal physiology of these target organs [71].

These adipokines modulate the expression of miRNAs involved in adipogenesis [50]. Xu et al. demonstrated that leptin increased the expression of miR-378 in human adipocytes [72]. Data from the literature clearly depicts miRNAs as important for the secretion or the effect of adipokines (leptin, adiponectin) (Figure 1). For example, miR-218 can target the 3′UTR of the mRNA coding for the adiponectin receptor (AdipoR), which inhibits the effect of adiponectin on glucose uptake [73]. At the central level, several miRNAs are identified as targets of leptin in the context of feeding behavior [74,75,76,77,78]. Conversely, the group of M. Taouis demonstrated that overexpression of miR-200a in the hypothalamus of ob/ob mice can down-regulate leptin receptor hypothalamic expression [76]. In a recent paper, our group exhibited that Dicer-derived miRNAs may be involved in the hypothalamic sensitivity of leptin [79].

### 2.4. The Inflammation of the Adipose Tissue and MiRNAs

It is well known that chronic inflammation is a key feature of obesity, and this obesity-associated inflammation is involved in the onset of the metabolic syndrome [80,81]. A number of miRNAs act as regulators of the expression of inflammatory markers [40,82,83] (Figure 1). For instance, the over-expression of miR-132 in primary human adipose-derived stem cells leads to an increase in the production of interleukin-8 and monocyte chemoattractant protein-1 (MCP1) [84]. It has also been shown that miR-126 and miR-193b regulate the expression and the release of MCP1, respectively [85]. Another set of studies revealed that miRNAs can modulate the expression and/or the secretion of TNF-α. More precisely, the decrease in the expression of miR-221 is associated with a high level of TNF-α in the human adipose tissue-derived mesenchymal stem cells from obese woman [86]. Lorente-Cebrian et al. observed that miR-145 stimulates the expression of TNF-α in adipocytes through the activation of the nuclear transcription factor kappa-B (NF-κB) pathway [87]. Interestingly, our group established a proinflammatory connection between NF-κB and miR-155 that could participate in the amplification of inflammatory status in adipocytes [88].

Several studies have suggested a dysregulation of miRNAs expression during the inflammation of the adipose tissue. The profile of expression of miRNAs is similar in the TNF-α-treated adipocyte 3T3-L1 cell line and in the adipocytes from the leptin deficient ob/ob mice model [89]. In human preadipocyte, TNF-α or leptin induce a decrease of miR-221 and an increase of miR-335 [86,90]. In the murine adipocyte model, TNF-α leads to an increase in the expression of miR-130, miR-146a, miR-146b, miR-150, miR-221, miR-222 and the decrease of miR-103 and miR-143 levels [89,91,92,93].

Altogether, these different studies have established that the expression of miRNAs in adipose tissue can be modulated by inflammation and that miRNAs can be also involved in the inflammation process induced by adipose tissue during the onset of obesity (Figure 1). It is now well documented that these inflammatory processes are associated with the defect of glucose homeostasis that leads to T2D. Particularly, the pro-inflammatory factors can induce impairment of insulin secretion, as well as the sensitivity to insulin in peripheral organs. Recently, miRNAs have been considered as new actors in these pathophysiological aspects.

### 2.5. The Role of MiRNAs in Insulin Synthesis, Secretion and Sensitivity

**Figure 2:**
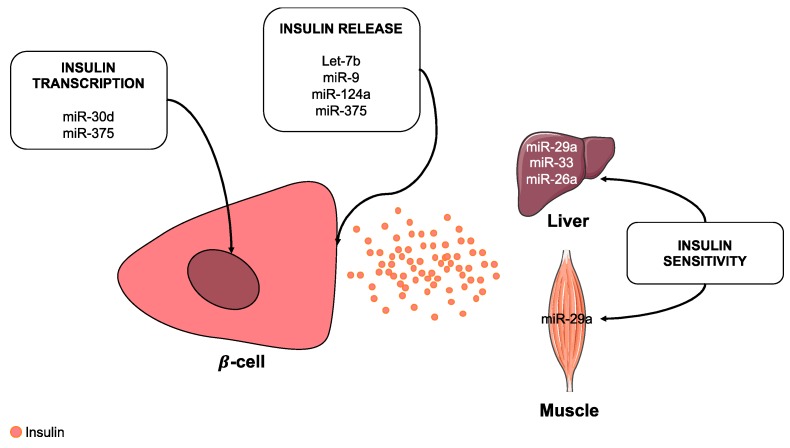
The miRNAs in the biosynthesis and effect of insulin. Several miRNAs are involved in the transcription and the release of insulin, as well as sensitivity to insulin in the liver and skeletal muscle.

T2D is characterized by a defect in insulin secretion, but also an impairment of insulin sensitivity. In this context, miRNAs play a role in insulin synthesis and secretion as well as in insulin sensitivity [94] (Figure 2). Different studies describe that ablation of Dicer in β-cell leads to the onset of diabetes because of impairment in glucose-stimulated insulin secretion (GSIS) and insulin biosynthesis [95,96,97]. Other studies have suggested that Ago is also crucial for the secretion of insulin [98]. Specific deletion of Ago in the β-cell line MIN6 enhances the biosynthesis of insulin [98]. Several miRNAs were originally identified to be expressed specifically in the pancreatic endocrine cell line [99]. Among them, the group of M. Stoffel described miR-375 as an important modulator of β-cell functions [100]. miR-375 overexpression in β-cells leads to a reduction of the number and viability of β-cells [100,101]. Interestingly, miR-375 regulates the release of insulin through direct targeting of genes such as apoptosis inducing factor mitochondria associated 1 (Aifm1), gephyrin (Gphn), tyrosine 3-monooxygenase/tryptophan 5-monooxygenase activation protein zeta (ywhaz) and myotrophin (MTPN) that are relevant for exocytosis [98,99]. It has also been suggested that miR-9 exerted an inhibitory effect on insulin exocytosis by an action on the transcription factor one cut homeobox 2 (Onecut2) and granuphilin (Sytl4), a negative regulator of secretion [102]. Additionally, miR-124a and *let-7b*, both of which are also abundantly expressed in pancreatic islet cells, are postulated to be important in the release of insulin through the modulation of MTPN expression [103].

As indicated above, miRNAs are also important actors in the transcription of insulin. Using the MIN6 cell line, Tang et al. highlighted that miR-30d is up-regulated in the presence of high glucose [104]. Interestingly, the overexpression of miR-30d increased insulin gene expression, while inhibition of miR-30d abolished glucose-stimulated insulin gene transcription [104]. The overexpression of miR-375 also suppressed glucose-stimulated insulin expression by targeting 3′-phosphoinositide-dependent protein kinase-1 (PDK1) indicating that miR-375 is one of most relevant miRNAs in pancreas [99,101].

The skeletal muscle and liver are the main users of glucose under the action of insulin (Figure 2). In these organs, several studies depicted mechanisms of insulin resistance involving miRNAs. Recently, Zhou et al. established that miR-29a was involved in insulin resistance by targeting PPARδ which led to a decrease in the levels of glucose transporter type 4 (GLUT4) in skeletal muscle [105]. Interestingly, several miRNAs are potentially involved in the expression of GLUT4 by targeting GLUT4 mRNA directly or indirectly by transcription factors such as peroxisome proliferator-activated receptor gamma coactivator 1-alpha (PGC1α) as well as Krüeppel-like factor 15 (KLF15) [106]. The importance of miR-29a in insulin resistance was confirmed by a study suggesting that miR-29a was up-regulated in the liver of db/db mice [107]. This increase in the expression of miR-29a was consequently accompanied by an attenuation of insulin inhibition of the expression of phosphoenol pyruvate carboxykinase (PEPCK) that is involved in the inhibition of gluconeogenesis [107]. In mice, a high fat diet increased the expression of miR-29a in myocytes, which led to an impairment in insulin signaling by decreasing insulin receptor substrate 1 (IRS1) expression [108]. In the liver, insulin resistance was also associated with miR-33, which modulates the expression of insulin receptor substrate 2 (IRS2) [109]. Interestingly, overweight humans had decreased expression of miR-26a in the liver compared to lean individuals [110]. In addition, liver-specific overexpression of miR-26a in mice fed a high-fat diet showed improved insulin sensitivity [110].

## 3. Conclusions

As illustrated in this review, miRNAs have emerged as key regulators of endocrine functions. Results from previous studies exhibit that miRNA expression is associated with different aspects of metabolic diseases: development of organs, release of hormones and sensitivity to hormones. We have also reported a potential role for miRNAs as biomarkers for the diagnosis or prognosis of different endocrine diseases. In our opinion, the most relevant finding in the field of miRNA research is the novel discovery that miRNAs packaged within cell-secreted exosomes could be critical for the cross-talk between different organs. In particular, recent studies demonstrated the role of exosomes in the communication between adipose tissue and the liver. Additional studies are required to entirely understand this novel mechanism of cell–cell communication.
